# Supplementary material for: The Transcriptome Landscape of the In Vitro Human Airway Epithelium Response to SARS-CoV-2
Source: Int J Mol Sci. 2023 Jul 27;24(15):12017. doi: 10.3390/ijms241512017 (PMC10418806; doi:10.3390/ijms241512017)
Supplement: Supplementary file 1 [file ijms-24-12017-s001.zip › ijms-2440321-supplementary/ijms-2440321-supplementary-final/Captions.pdf]

Figure S1: Mechanistic networks showing the interactions between up-stream regulators (TNF, IL1A, F2) and their target genes identified in the SARS-CoV-2 bulk RNA-seq signature. The IPA upstream regulator analysis is based on the expected causal effects between upstream regulators and targets;

Figure S2: The standard curves of genes GJA1, IRF9, OAS1 and CXCL10. The experiment was conducted using a 2-fold serial dilution on a cDNA pool (in triplicate). Data is presented as mean +/- SD;

Figure S3: Comparison of relative gene expression using RTqPCR in non-infected iALI samples (mock) at day 0 (n=3) and day 4 (n=1), as a control of Figure 4D. There is no significative change between day 0 and day 4.

Table S1: Primer pairs used for validation by RT-qPCR. When existing, the reference of the paper from which the sequence was found is cited;

Table S2: List of the 515 genes specific to the SARS-CoV-2 bulk RNA-seq signature (transcriptome analysis by Blanco-Melo, D et al. [18]);

Table S3: List of the top GO categories identified by Ingenuity Pathway Analysis using the 515 genes listed in Table S2;

Table S4: List of the 146 enriched canonical pathways identified in the SARS-CoV-2 bulk RNA-seq signature;

Table S5: Lists of genes targeted by the upstream regulator's TNF, IL1A and F2 identified using IPA Upstream Regulator Analysis;

Table S6: Exhaustive lists of miRNAs that are putative regulators of genes implicated in interferon and inflammatory responses upon SARS-CoV-2 infection of ALI cultures retrieved by GenGo Metacore.
