# Supplementary material for: The Transcriptome Landscape of the In Vitro Human Airway Epithelium Response to SARS-CoV-2
Source: Int J Mol Sci. 2023 Jul 27;24(15):12017. doi: 10.3390/ijms241512017 (PMC10418806; doi:10.3390/ijms241512017)
Supplement: Supplementary file 1 [file ijms-24-12017-s001.zip › ijms-2440321-supplementary/ijms-2440321-supplementary-final/Supplementary Table S1_List primers.pdf]

Table S1 :

Genes	Primer forward	Primer reverse	Paper reference (PMID)
**IFI35**	GAGGGTGTTGGTCACTGGATT	CA CCTCCGTTCCTAGTCTTGC	PMID: **28064541**
**IFI44**	TCCAAGGGCATGTAACGCAT	CCTCCCTTAGATTCCCTATTTGCT	PMID: **34760904**
**IFIT1**	GCCTTGCTGAAGTGTGGAGGAA	ATCCAGGCGATAGGCAGAGATC	PMID : **33142113**
**IFIT3**	GAAACAGCCATCATGAGTGAGG	GCATCTGAGAGTCTGCCCAA	PMID: **34760904**
**ITGB1**	TGATTGGCTGGAGGAATGTTA	GTTTCTGGACAAGGTGAGCAA	PMID: **33345684**
**MX1**	GGCTGTTTACCAGACTCCGACA	CACAAAGCCTGGCAGCTCTCTA	PMID: **33142113**
**OAS3**	GAAGGAGTTCGTAGAGAAGGCG	CCCTTGACAGTTTTCAGCACC	PMID: **32321151**
**ISG15**	GCAGATCACCCAGAAGATCG	GGCCCTTGTTATTCCTCACC	PMID: **35637255**
**SARS-CoV-2 envelope (gene E)**	ACAGGTACGTTAATAGTTAATAGCGT	ATATTGCAGCAGTACGCACACA	PMID: **31992387**
**GAPDH**	CCACCTTTGACGCTGGG	CATACCAGGAAATGAGCTTGACA	PMID : **15331414**
**GJA1**	GGCCTTCTTGCTGATCCAGT	GCTGGTCCACAATGGCTAGT	De novo primer
**IRF9**	TATCAGTTGCTGCCACCAGG	TAGGATGCCCCTCTCAAGCT	
**OAS1**	CCTACCCTGTGTGTGTGTCC	GCATAGACCGTCAGGAGCTC	
**CXCL10**	CCTCCAGTCTCAGCACCATG	TGAAGCAGGGTCAGAACATCC	
