# Supplementary figures and images for: The Transcriptome Landscape of the In Vitro Human Airway Epithelium Response to SARS-CoV-2
Source: Int J Mol Sci. 2023 Jul 27;24(15):12017. doi: 10.3390/ijms241512017 (PMC10418806; doi:10.3390/ijms241512017)

## Slide 1
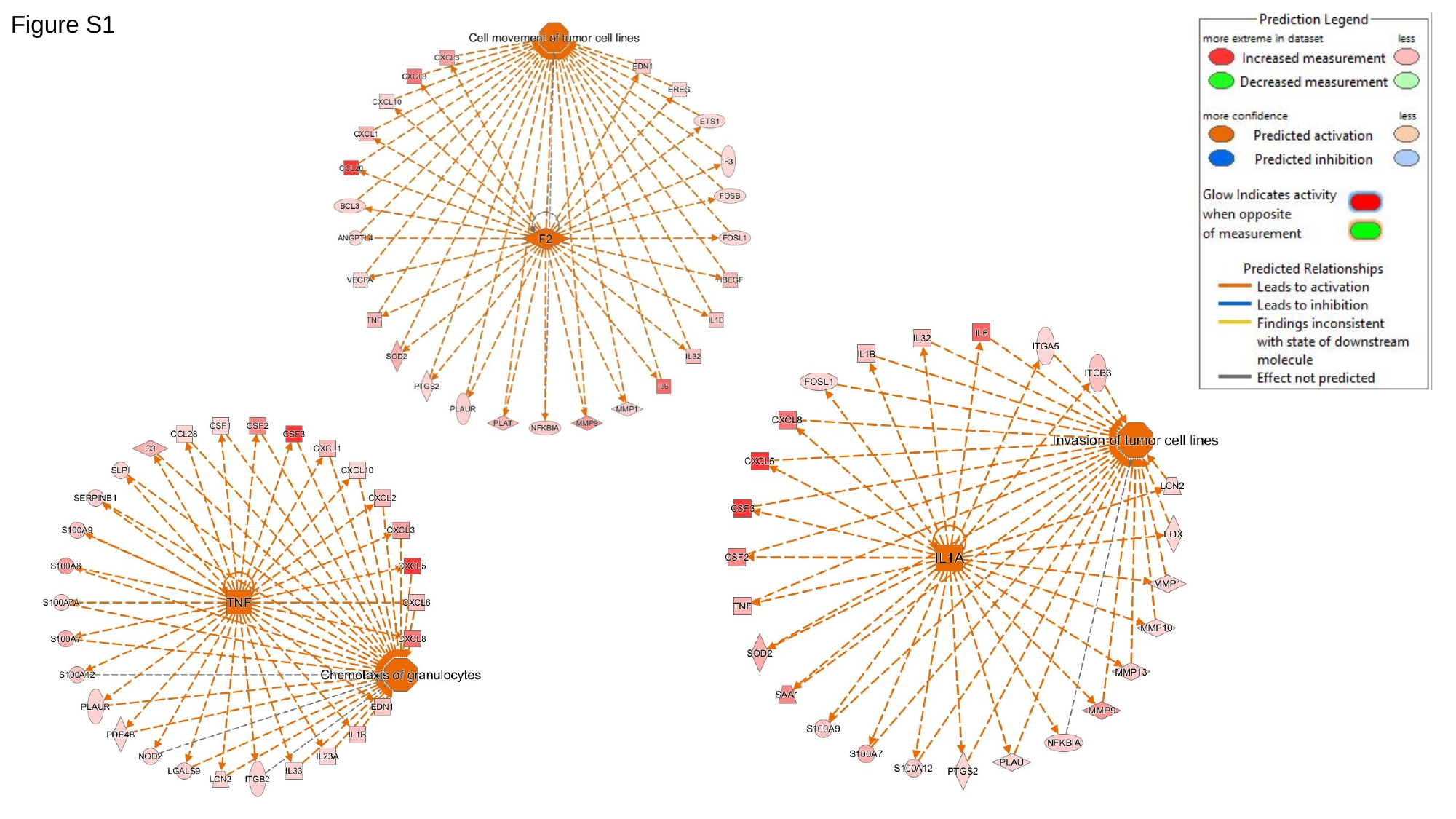

Figure S1

Supplement: Supplementary file 1 [file ijms-24-12017-s001.zip › ijms-2440321-supplementary/ijms-2440321-supplementary-final/Supplementary Figure S1_upstream regulators_IPA.pptx]

## Slide 1
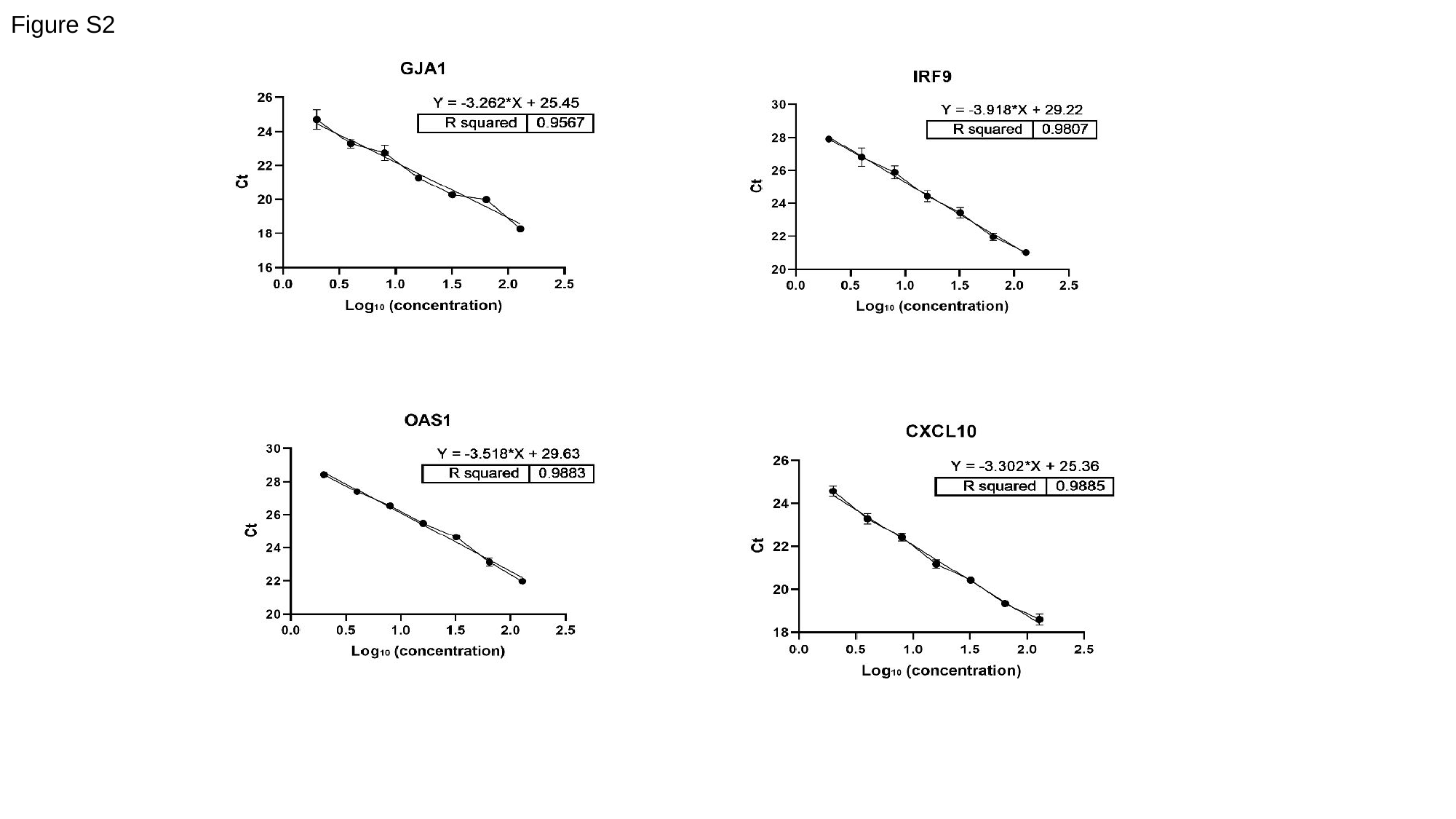

Figure S2

Supplement: Supplementary file 1 [file ijms-24-12017-s001.zip › ijms-2440321-supplementary/ijms-2440321-supplementary-final/Supplementary Figure S2_RTqPCR primers sequence and validation.pptx]

## Slide 1
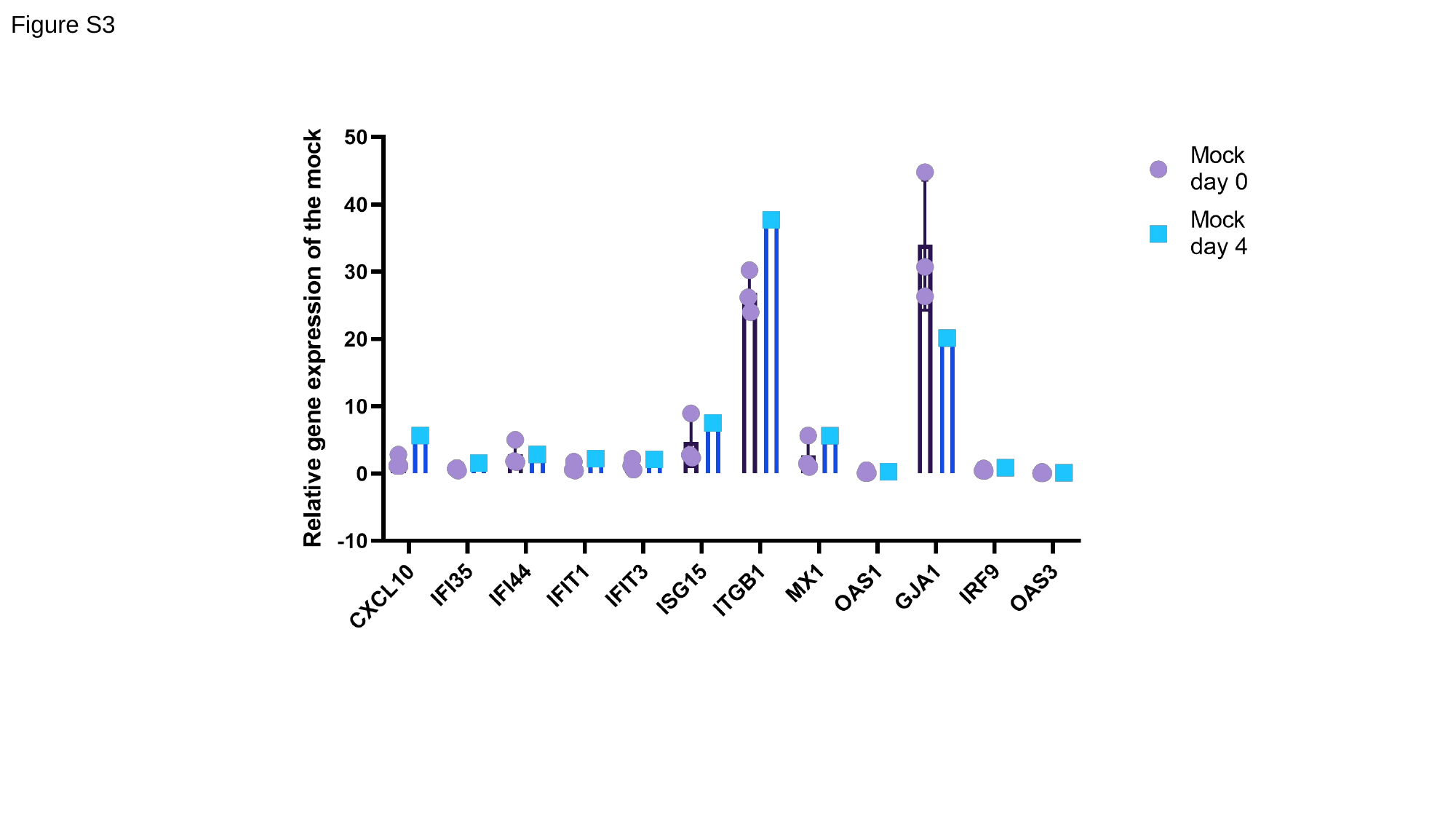

Figure S3

Supplement: Supplementary file 1 [file ijms-24-12017-s001.zip › ijms-2440321-supplementary/ijms-2440321-supplementary-final/Supplementary Figure S3_Mock control.pptx]
